# CNOT6 regulates a novel pattern of mRNA deadenylation during oocyte meiotic maturation

**DOI:** 10.1038/s41598-018-25187-0

**Published:** 2018-05-01

**Authors:** Karl-Frédéric Vieux, Hugh J. Clarke

**Affiliations:** 1 Department of Biology, McGill University, Montreal, Quebec Canada; 2 Department of Obstetrics and Gynecology, McGill University, Montreal, Quebec Canada; 3 Research Institute of the McGill University Health Centre, Montreal, Quebec Canada

## Abstract

In many cell types, the length of the poly(A) tail of an mRNA is closely linked to its fate - a long tail is associated with active translation, a short tail with silencing and degradation. During mammalian oocyte development, two contrasting patterns of polyadenylation have been identified. Some mRNAs carry a long poly(A) tail during the growth stage and are actively translated, then become deadenylated and down-regulated during the subsequent stage, termed meiotic maturation. Other mRNAs carry a short tail poly(A) tail and are translationally repressed during growth, and their poly(A) tail lengthens and they become translationally activated during maturation. As well, a program of elimination of this ‘maternal’ mRNA is initiated during oocyte maturation. Here we describe a third pattern of polyadenylation: mRNAs are deadenylated in growing oocytes, become polyadenylated during early maturation and then deadenylated during late maturation. We show that the deadenylase, CNOT6, is present in cortical foci of oocytes and regulates deadenylation of these mRNAs, and that PUF-binding elements (PBEs) regulate deadenylation in mature oocytes. Unexpectedly, maintaining a long poly(A) tail neither enhances translation nor inhibits degradation of these mRNAs. Our findings implicate multiple machineries, more complex than previously thought, in regulating mRNA activity in oocytes.

## Introduction

A hallmark of germ cell differentiation is the central role played by post-transcriptional mechanisms in regulating gene expression. In female vertebrates, post-natal development of the oocyte comprises two phases – a prolonged growth phase during which the oocyte increases enormously in size, and a much shorter phase termed meiotic maturation during which it completes the first meiotic division^1,2^. During growth, oocytes synthesize large quantities of mRNA that owing to its extraordinary stability – its half-life in mice is estimated to be 3 weeks^3^ – accumulates to form a large stock of ‘maternal’ mRNA. Some of the accumulated mRNA species are immediately translated to support the growth process, whereas other species are stored in ribonucleoprotein particles in a translationally inactive form. When fully grown oocytes enter meiotic maturation, however, the situation changes dramatically – many previously active mRNAs become translationally silenced, whereas previously silent mRNAs become activated. In addition, through a process that is incompletely understood, the stock of maternal mRNA begins to be degraded. Thus, both the translational activity and the stability of mRNAs are dynamically regulated during oocyte growth and meiotic maturation^4–7^.

In many cell types, changes in mRNA translation and stability are closely linked to changes in the length of the poly(A) tail, a sequence of adenine residues that is added to the 3′-end of most mRNAs co-transcriptionally in the nucleus^6,8–10^. The poly(A) tail provides a docking site for the cytoplasmic poly(A) binding protein (PABPC), which together with the translation initiation factor eIF4G and the cap-binding protein eIF4E promote translation. A long poly(A) tail is also associated with mRNA stability; in contrast, a short poly(A) is associated with translational inactivity and degradation. Following the export of polyadenylated mRNAs to the cytoplasm, competing activities of poly(A) polymerases and deadenylases respectively lengthen or shorten the poly(A) tail. Lengthening of the poly(A) tail is promoted by coordinated activity of the cleavage and polyadenylation specificity factor (CPSF), which binds to the polyadenylation signal (AAUAAA) located near the 3′-end of the mRNA, and poly(A) polymerases, which catalyse the polyadenylation reaction. Shortening of the tail is promoted by deadenylases, of which two major cytoplasmic families have been identified^11–13^. The poly(A)-specific nuclease (PAN), comprising the PAN2 catalytic subunit in a complex with PAN3 is thought to be responsible at least in some cell types for the initial deadenylation of an mRNA^14,15^. The major deadenylase activity in most cell types, however, is conferred by the CCR4-NOT complex. This multi-protein subunit consists of five or more main subunits, including NOT family members that provide structural integrity to the complex and two proteins that possess deadenylase activity – one of either CNOT6 or CNOT6L, members of the EEP (exonuclease-endonuclease-phosphatase) family of deadenylases, and one of either CNOT7 or CNOT8, members of the DEDD family of deadenylases that are named for the conserved Asp and Gln in their catalytic domains. Although both EEP and DEDD members are catalytically active, they can target different mRNAs^11,16,17^. This provides a potential mechanism for selective deadenylation of specific mRNA species, although the mechanism underlying the selectivity remains to be identified. Both CCR4-NOT and PAN2/3 can be recruited to the poly(A) tail of mRNAs, thus mechanistically coupling deadenylation to prior polyadenylation.

Two patterns of mRNA polyadenylation dynamics have been identified in oocytes^4–6^. Many mRNAs carry a long poly(A) tail in growing and fully grown immature oocytes and are actively translated. During maturation, however, these mRNAs become deadenylated and their translational activity decreases. In contrast, other mRNAs carry a short poly(A) tail in growing and fully grown immature oocytes and are only weakly translationally active; during maturation, the poly(A) tail of these mRNAs lengthens and they become translationally active. Deadenylation of mRNAs during maturation has also been linked to their degradation. The pattern of polyadenylation of an mRNA is strongly influenced by sequences in its 3ʹ-untranslated region (UTR), including a U-rich sequence termed the cytoplasmic polyadenylation element (CPE), the PUF-binding element (PBE), and the Musashi-binding element^6,18–22^. In particular, the presence or absence, respectively, of a CPE within about 200 nt of the polyadenylation signal frequently dictates whether an mRNA will become polyadenylated or deadenylated during maturation. These complementary patterns of polyadenylation dynamics suggest a simple dichotomous model in which two classes of oocyte mRNA exist in maturing oocytes – one becoming polyadenylated and translationally active, and the other becoming deadenylated, translationally silent, and degraded.

Emerging evidence now indicates that the translational landscape during meiotic maturation is more complex than originally anticipated, as exemplified by changes in the translational activity of different mRNAs at different stages of maturation^20,23–25^ and the selective degradation of some mRNAs while others remain stable until after fertilization^3,26^. This raises the possibility that mRNA polyadenylation dynamics and function during maturation might be more varied than implied by the dichotomous model. Here we identify a oocyte mRNAs which display a novel pattern of adenylation during maturation. These mRNAs carry a short poly(A) tail in growing oocytes, become polyadenylated during the early portion of oocyte maturation, and then are deadenylated during late maturation coincident with partial degradation. The CNOT6 deadenylase selectively regulates the deadenylation of these mRNAs, and we uncover a role for specific sequences in the 3′-UTR of the mRNAs in mediating deadenylation. Surprisingly, preventing their deadenylation during late maturation by depletion of *Cnot6* neither increases their translation nor prevents their degradation. These results indicate that the multiple patterns of mRNA adenylation co-exist in maturing oocytes and in addition suggest that poly(A) tail length may not play a deterministic role in regulating mRNA translation and stability in these cells.

## Results

### Identification of a novel pattern of mRNA adenylation during oocyte maturation

To study the relationship between the poly(A) and the activity of an mRNA, we used a method termed RL-PAT that we and others have developed to measure poly(A) tail-length^27–31^. Briefly, an RNA linker is ligated to the 3′-end of the mRNA, which is then subjected to RT-PCR using gene- and linker-specific primers. This generates PCR products of a single size, in contrast to the multiple lengths produced using methods based on oligo-dT, which may be able to anneal at many sites on the poly(A) tail. We focussed on the poly(A) tail dynamics of three mRNAs known to become translationally activated during meiotic maturation: *Ccnb1*, which encodes cyclin B1; *Orc6*, which encodes a DNA replication factor; and *Slbp*, which encodes a protein that binds to a stem-loop structure in the 3′-untranslated region (UTR) of histone mRNAs^32,33^. Maternal cyclin B1 is required for the completion of meiotic maturation, whereas maternal ORC6 and SLBP are required for embryonic development.

**Figure 1:**
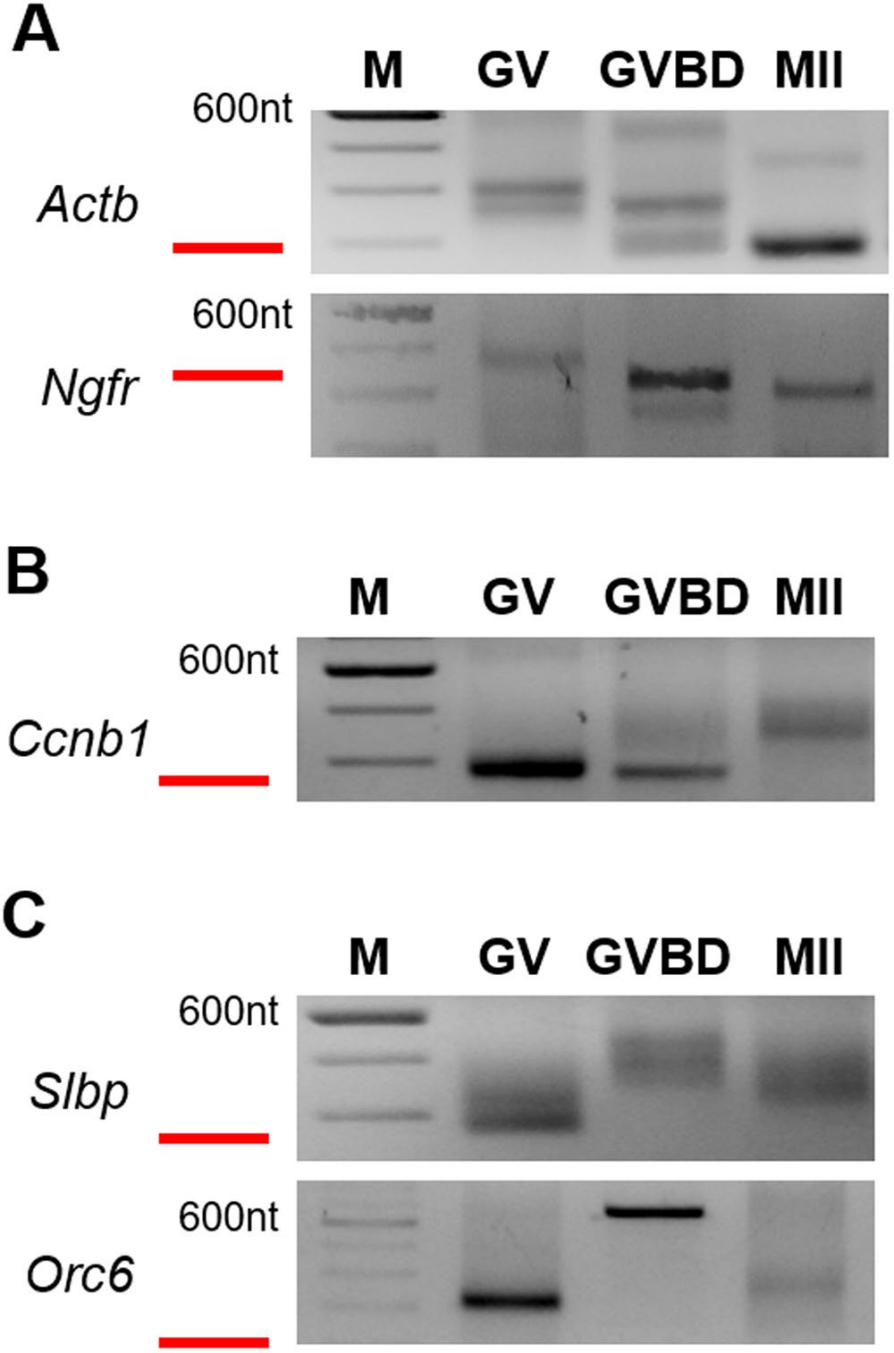
Patterns of mRNA polyadenylation during oocyte meiotic maturation. Immature oocytes containing an intact germinal vesicle (GV) were obtained from antral follicles and collected immediately (GV), or allowed to mature for 6 hr (germinal vesicle breakdown, GVBD) or 12–15 hr (metaphase II, MII). Poly(A) tail length of the indicated mRNAs was analyzed used the RL-PAT technique. Red bars indicate the product length that would be generated using a non-adenylated mRNA. Molecular weight markers (100 bp ladder) are shown in left-most lane. (**A**) *Actb* and *Ngfr* become progressively deadenylated. (**B**) *Ccnb1* becomes progressively polyadenylated. (**C**) *Slbp* and *Orc6* become polyadenylated and subsequently deadenylated.

Consistent with previous studies, the poly(A) tail of mRNAs that are translated in immature oocytes shortened during maturation (Fig. 1A – *Actb*, *Ngfr*), whereas that of *Ccnb1* lengthened (Fig. 1B). Unexpectedly, however, we observed a third pattern of polyadenylation for *Orc6* and *Slbp*. Both mRNAs carried a short poly(A) tail in immature oocytes, which lengthened substantially during the early portion of maturation, following germinal vesicle breakdown (GVBD). These results are consistent with previous data showing a putative CPE in the 3′-UTR of each mRNA and the accumulation of both proteins during maturation^32,34,35^. As maturation progressed to metaphase II, however, the poly(A) tail of both mRNAs subsequently shortened (Fig. 1C, MII group)^27^. Thus, at least three different patterns of mRNA polyadenylation occur during oocyte maturation.

### CNOT6 is expressed in immature and maturing oocytes

**Figure 2:**
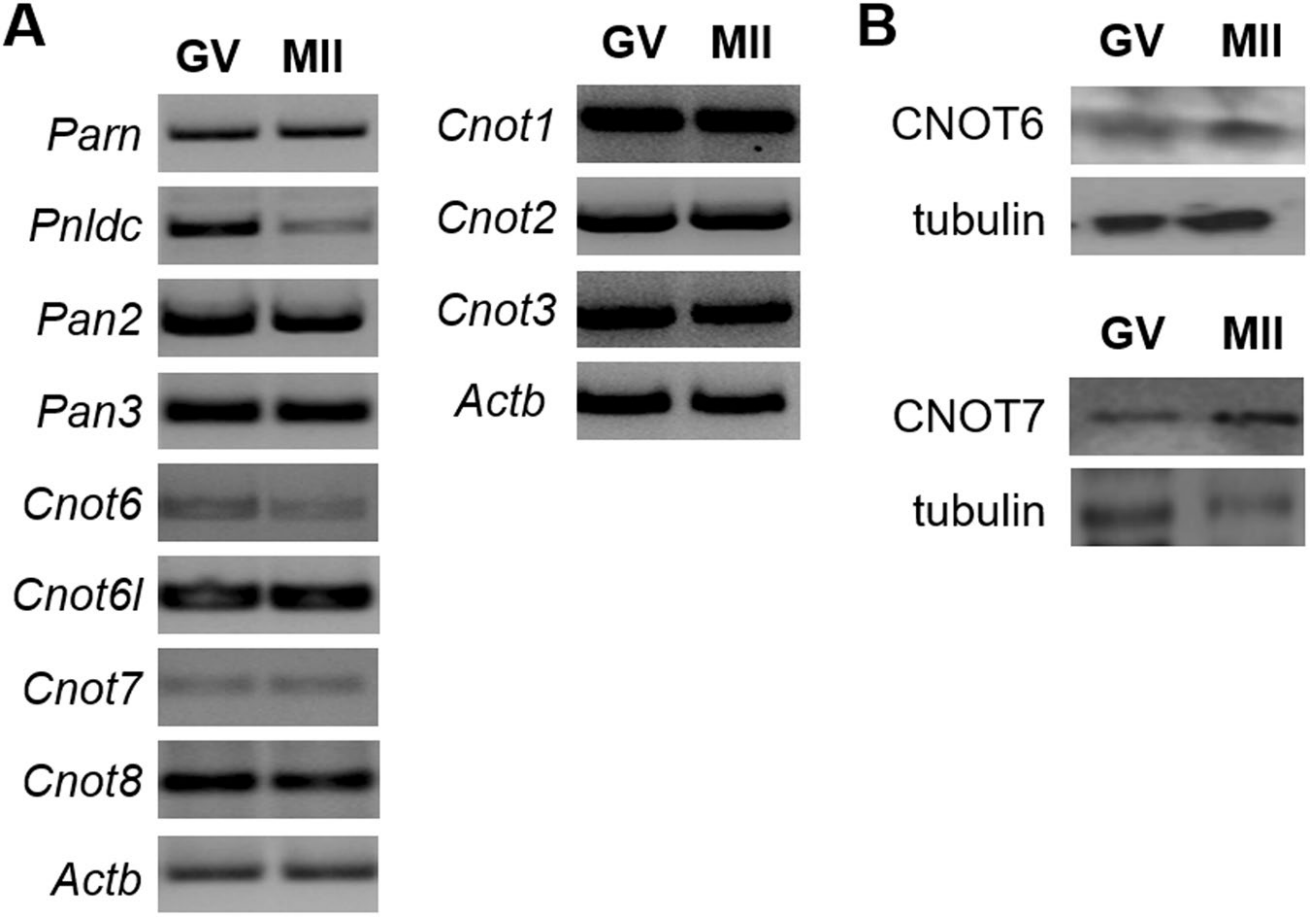
Expression of deadenylases in oocytes. Oocytes were obtained as in Fig. 1. (**A**) mRNAs were assayed using the polymerase chain reaction and the primers shown in Table 2. mRNAs encoding all major deadenylases are present in both immature (GV) and mature (MII) oocytes. *Actb* is used as a loading control. (**B**) CNOT6 and CNOT7 protein were assayed by immunoblotting using commercially available antibodies. Panels shows representative blots.

To gain insight into the mechanism of these diverse patterns of adenylation, we began by identifying deadenylases expressed by oocytes, as previous results indicated that deadenylases can display mRNA-specificity^3,36^. We found that oocytes express mRNAs encoding a wide range of CNOT family members, including its catalytic subunits encoded by *Cnot6*, *Cnot6l*, *Cnot7* and *Cnot*8; they also expressed *Pan2* and *Pan3*, the nuclear deadenylase, *Parn*, and the related deadenylase, *Pnldc* (Fig. 2A). Using available antibodies, we confirmed that CNOT6 is present in both immature and maturing oocytes (Fig. 2B). CNOT7 is also expressed, confirming previous reports^3,36^. Because it is thought to target different mRNAs than those regulated by CONT7, we focused our studies on CNOT6.

**Figure 3:**
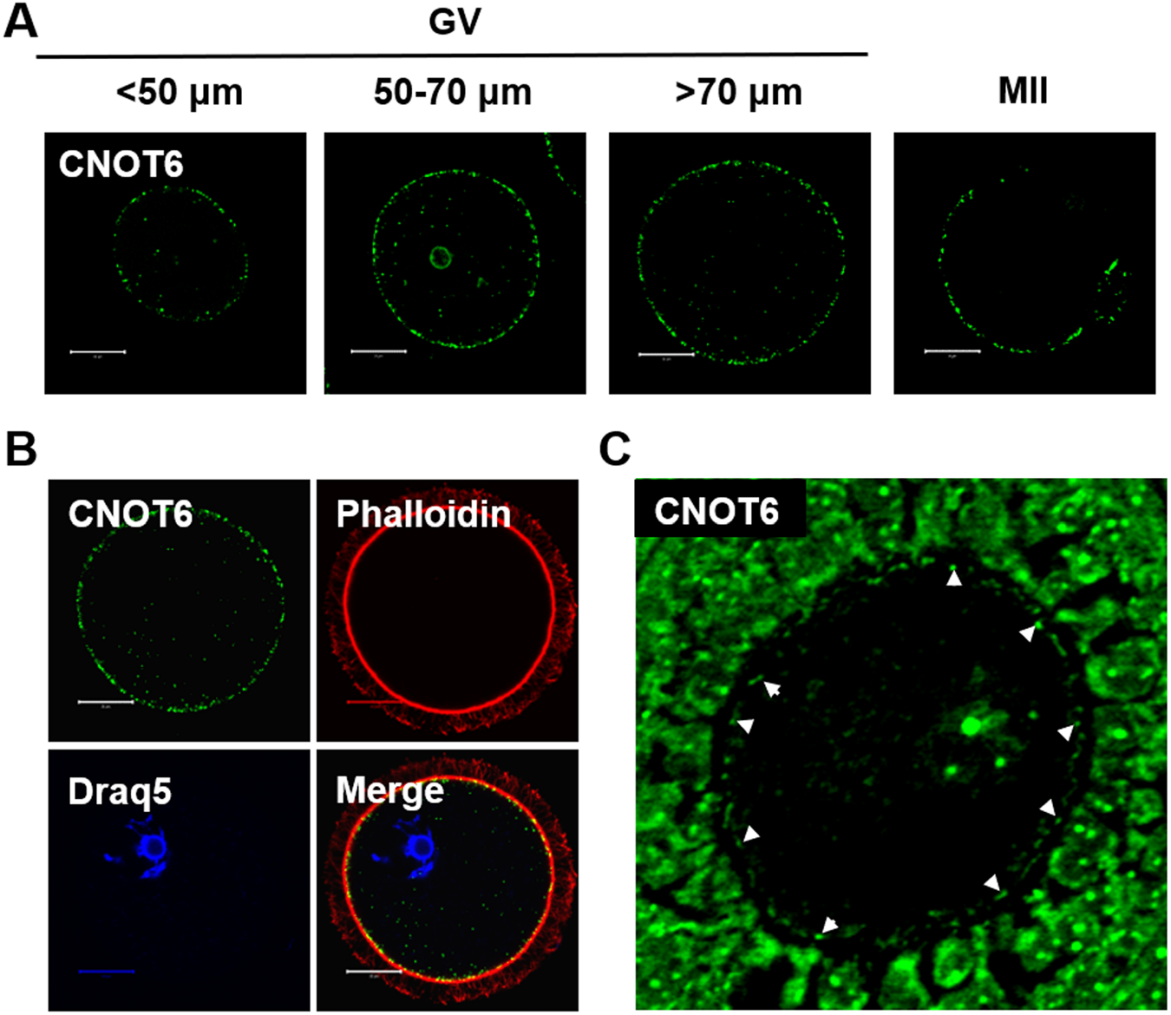
Localization of CNOT6 in growing and fully grown oocytes. (**A**) Whole-cell immunofluorescence of growing, fully grown and mature (MII) oocytes. Foci of CNOT6 staining are detectable at the periphery of the oocyte. (**B**) Whole-cell immunofluorescence of fully grown oocytes co-stained using anti-CNOT6 (green) and phalloidin (red) to reveal the actin-rich cortex. CNOT6 foci are predominantly located within the cortex of the oocytes. The nucleus is labelled using DRAQ5 (blue). (**C**) Immunohistochemistry of sections of paraffin-embedded ovaries confirms the cortical distribution of CNOT6 foci (arrowheads) in oocytes. Bar = 20 µm.

In somatic cells, CNOT6 accumulates in cytoplasmic ribonucleoprotein particles (RNPs) that contain mRNAs and proteins implicated in mRNA processing^37–39^. RNPs sharing a similar molecular composition have been identified in mouse oocytes, where they are localized in the cortical region^40–42^, and so we examined the distribution of CNOT6 in oocytes. We collected growing and fully grown immature oocytes and mature oocytes, and examined CNOT6 localization using immunofluorescence. We observed a narrow band of prominent foci of staining located near the periphery of the oocytes at all three stages of development (Fig. 3A). A small number of foci were also apparent more centrally within the cytoplasm. To accurately establish the intracellular location of the prominent foci, we used phalloidin to label the actin-rich cortex (Fig. 3B). This revealed that the foci lie within the cortical region of the oocyte. To verify this distribution, we also stained histological sections of ovaries which revealed, as observed using the whole-mount specimens, brightly stained CNOT6 foci at the periphery of oocytes (Fig. 3C).

**Figure 4:**
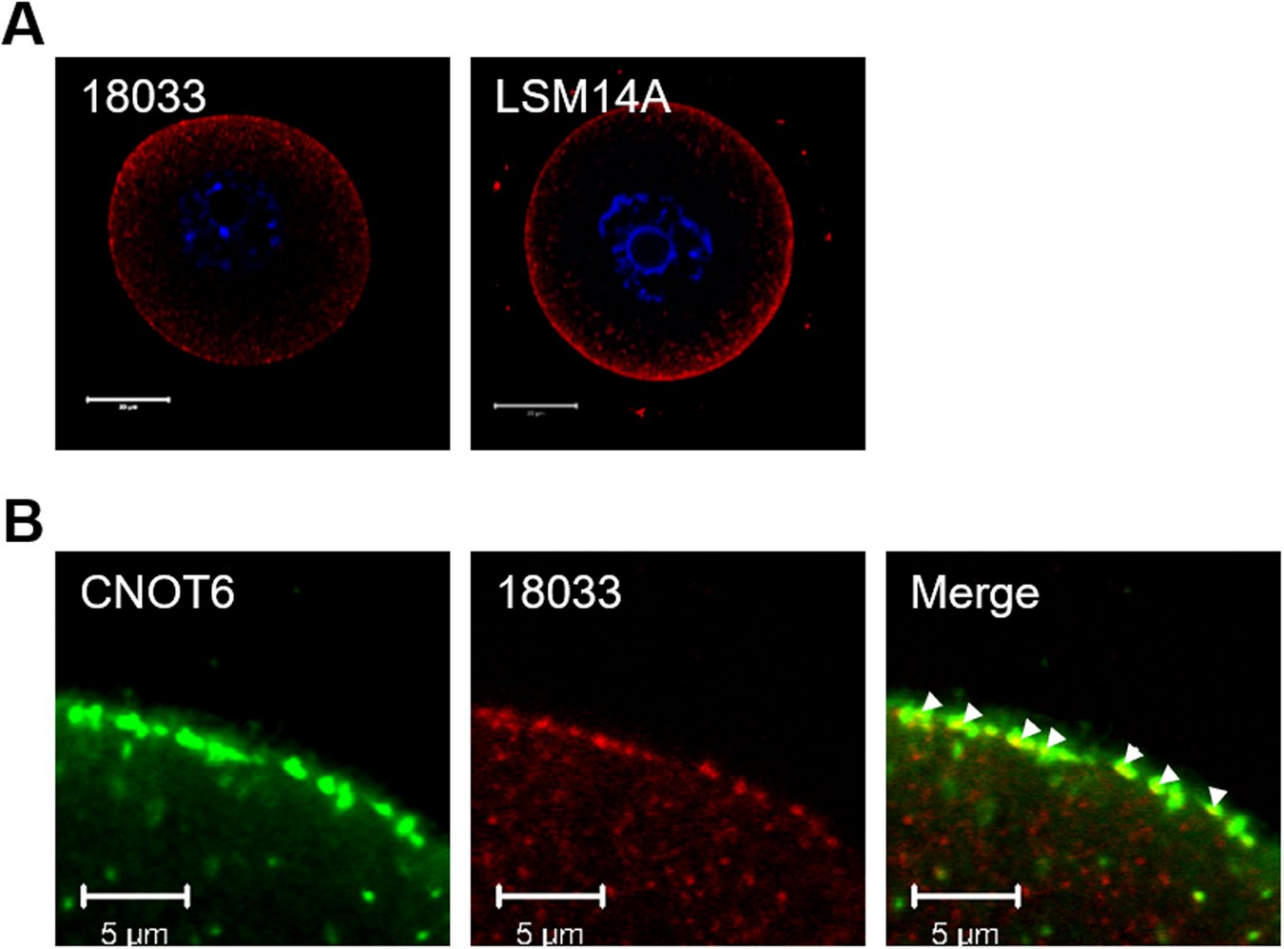
Co-localization of CNOT6 foci and cytoplasmic ribonucleoprotein (RNP) markers. (**A**) Whole-cell immunofluorescence of fully grown GV-stage oocytes stained for RNP markers 18033 (left panel) or LSM14A (right panel). Both are localized in the cortex. Bar = 20 µm. (**B**) Whole-cell immunofluorescence of fully grown GV-stage oocytes co-stained for CNOT6 (green) and 18033 (red). Merged image shows overlapping foci. Bar = 5 µm. Overlap was quantified using ImageJ (R = 0.73 ± 0.02, Rr = 0.32 ± 0.02; n = 12).

We then tested whether the CNOT6 foci might be related to the previously described cortical RNPs. TNRC6 (GW182) and EDC4, which are recognized by antiserum 18033, and the RNA-binding protein, LSM14, are components of RNPs known as P-bodies^41,43–45^. Antiserum 18033 and anti-LSM14 antibodies each stained the cortex of oocytes as previously reported^40,41^ and displayed a pattern remarkably similar to that observed for CNOT6 (Fig. 4). Because the CNOT6 antibody and serum 18033 are derived from different host species (rabbit and human, respectively), we were further able to test whether these proteins are co-localized. We found that the signals overlapped in many although not all cases (Fig. 4B). Two statistics were calculated to better evaluate this overlap: Mander’s overlap coefficient, which represents the fraction of pixels positive for both signals; and Pearson’s correlation coefficient, which represents the correlation in the fluctuation of the two signals from pixel to pixel^46^. Quantitative analysis of the staining patterns at the cortex of oocytes revealed a Mander’s overlap coefficient of 0.73 ± 0.02 (12 oocytes analysed), indicating a significant colocalization between CNOT6 and 18033 at the cortex, and a Pearson’s correlation coefficient of 0.32 ± 0.02 (12 oocytes analysed) indicating a positive linearity in the incidence of the two signals. Taken together, these results indicate that the cortical CNOT6 is present in structures that share characteristics with P-bodies.

### CNOT6 selectively deadenylates specific mRNAs

**Figure 5:**
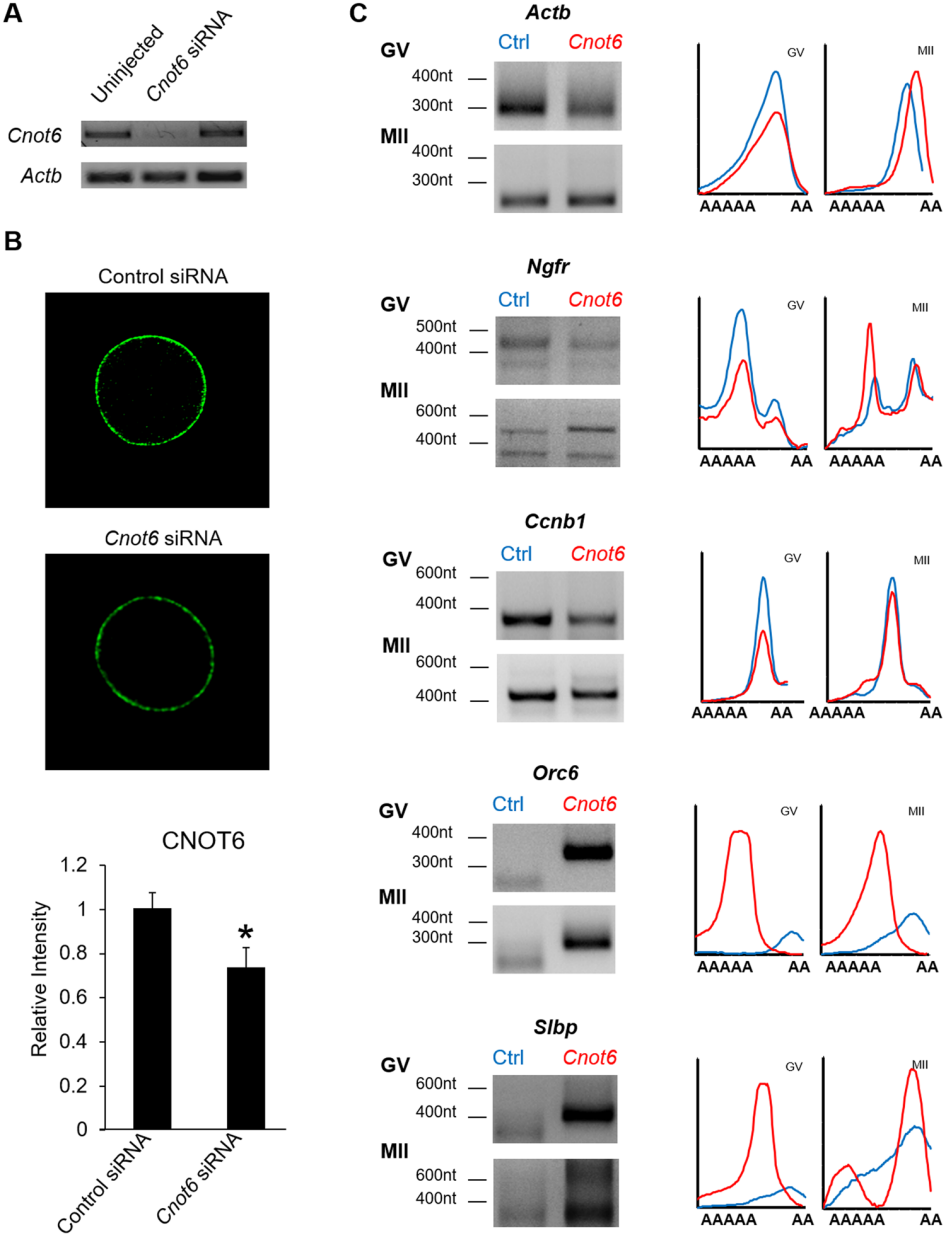
Reduction of CNOT6 impairs the deadenylation of *Slbp* and *Orc6* during late maturation. (**A**) Fully grown immature oocytes were injected with siRNA targeting *Cnot6* or a control or were left uninjected. The following day, oocytes were collected and *Cnot6* and *Actb* mRNA were assessed using RT-PCR. (**B**) Upper: Oocytes were injected as above, incubated for 24 hr, then stained using anti-CNOT6. Lower: Quantification of cortical fluorescence in oocytes injected with a control (39 oocytes) or *Cnot6* siRNA (41 oocytes). Oocytes used for quantification were collected in 3 independent experimental replicates. Asterisk indicates *p* < 0.05. (**C**) Fully grown immature oocytes were injected with the indicated siRNA and collected for analysis at the GV stage or after maturation to metaphase II (MII). RL-PAT was used to assess the poly(A) tail length of the indicated mRNAs. Left side shows representative images of gels after electrophoretic separation of PCR products. Right side shows quantification of gel from top (longer poly(A) tail) to bottom. *Cnot6*-siRNA injected oocytes shown in red, control siRNA-injected oocytes in blue. The experiment was repeated 3 times.

Having established that CNOT6 is expressed in oocytes, we then tested whether it regulates mRNA poly(A) tail length. We injected RNAi targeting *Cnot6* or a control into fully grown immature oocytes, incubated them overnight, then either continued to maintain them at the GV stage or allowed them to undergo maturation (Fig. 5A). By 24 hr after injection, both *Cnot6* mRNA and CNOT6 in cortical foci had been significantly depleted (Fig. 5B). Analysis using RL-PAT revealed no effect of CNOT6 depletion on the poly(A) tail of *Actb* and *Ngfr*, which becomes deadenylated during maturation, or of *Ccnb1*, which becomes stably polyadenylated during maturation (Fig. 5C). In striking contrast, however, CNOT6 depletion significantly affected the poly(A) tail of *Orc6* and *Slbp*. In both immature and mature oocytes, the length of the poly(A) tail was increased by about 100 nt, as compared to oocytes injected with a control RNAi (Fig. 5C). Thus, CNOT6 selectively regulates the poly(A) tail length of specific mRNAs.

### PUF-binding elements (PBE) are required for deadenylation during late maturation

**Figure 6:**
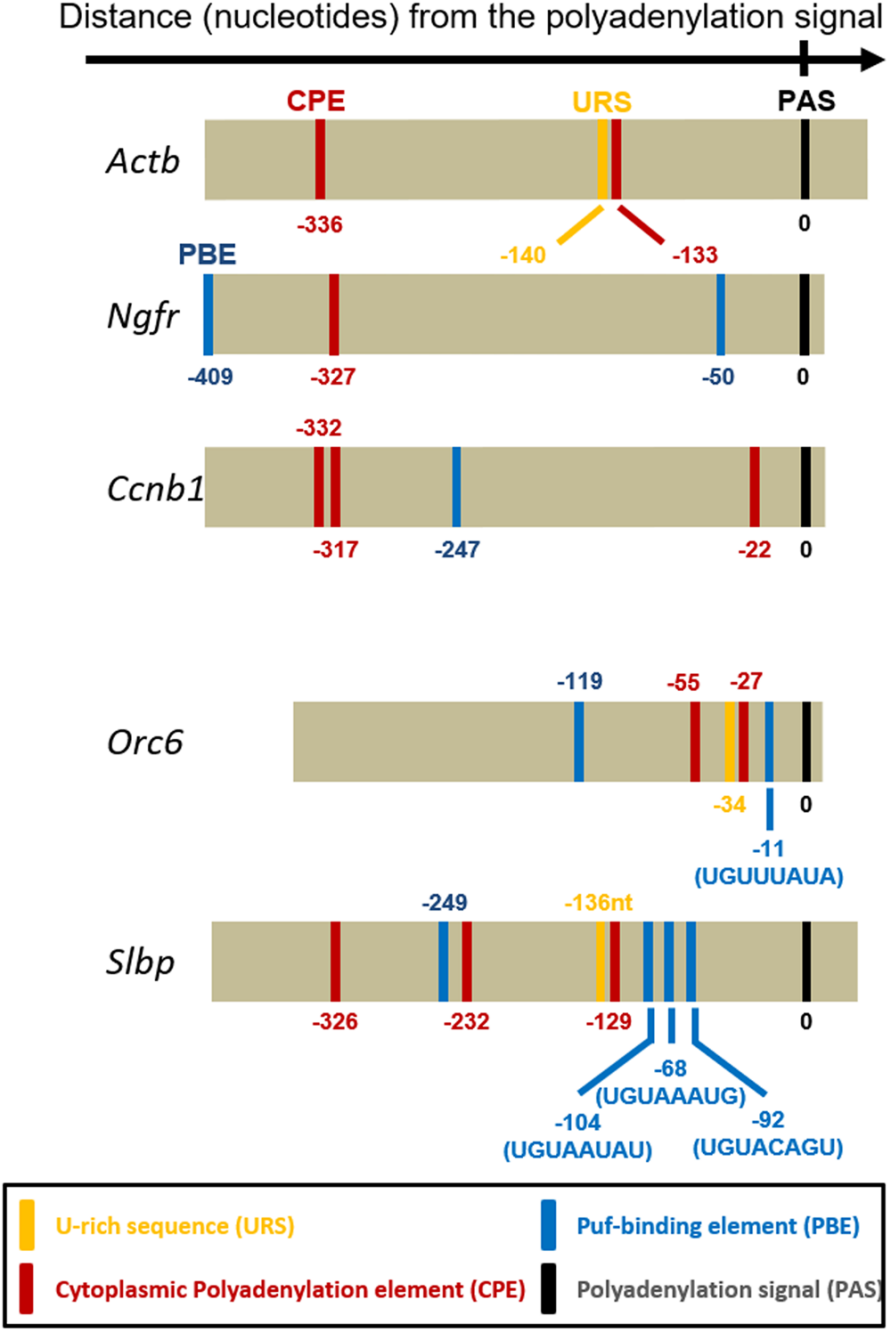
Terminal portion of the 3**′**-UTR of different mRNAs showing location of sequences that potentially regulate polyadenylation. Locations are indicated relative to the polyadenylation signal (PAS, black bar; sequence: AUUAAA). *Orc6* and *Slbp* mRNAs contain one or more potential PBE sequences (blue bars) located between the CPE (red bar) lying closest to the PAS and the PAS itself, whereas *Actb*, *Ngfr* and *Ccnb1* do not.

**Figure 7:**
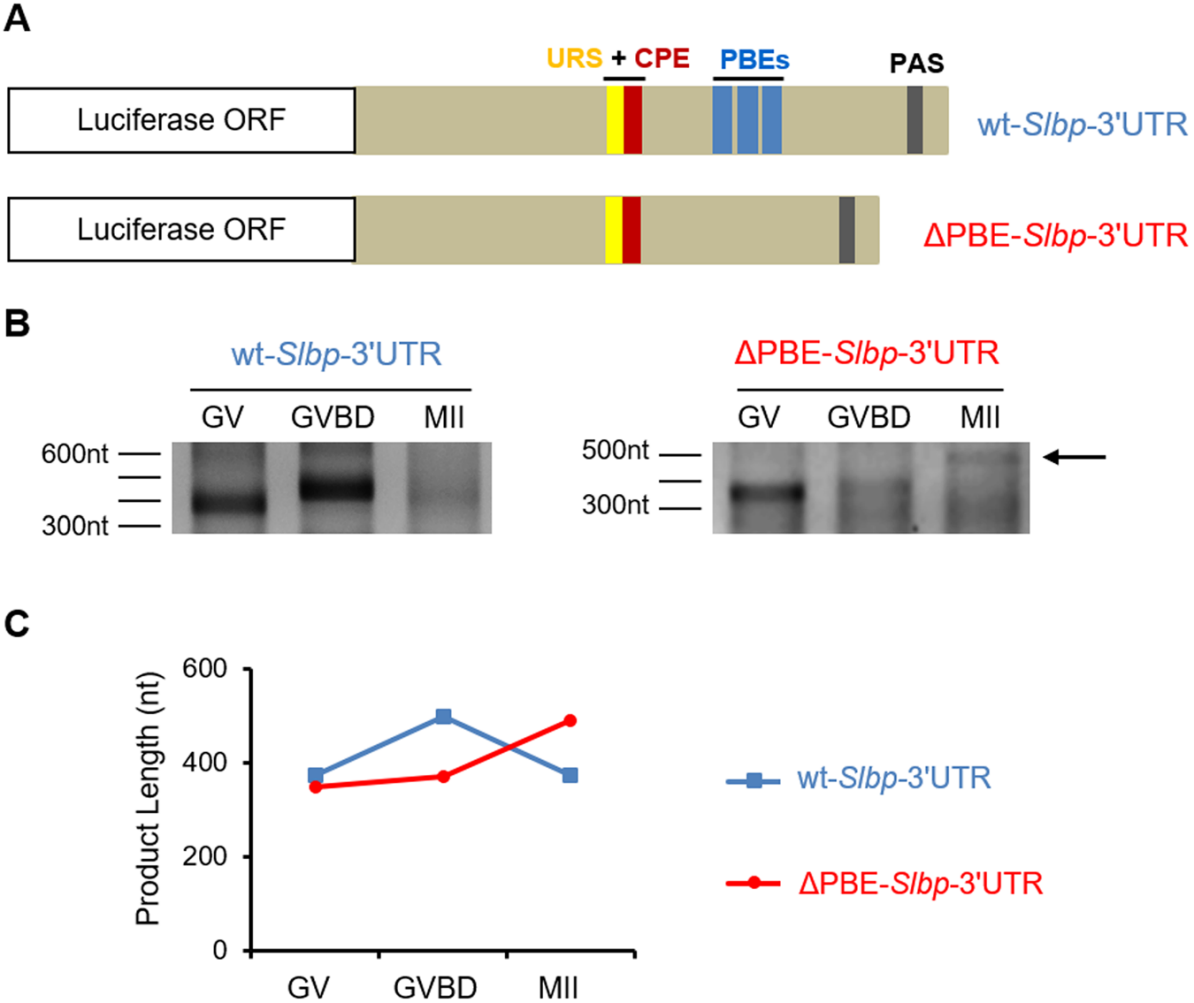
Luciferase-*Slbp* 3**′**-UTR constructs and injections. (**A**) The luciferase ORF was ligated either to the wild-type *Slbp-*3**′**UTR (upper construct) or to a mutant *Slbp* 3′UTR lacking the three putative PBE sequences downstream of the URS-CPE (lower construct). (**B**) mRNA synthesized *in vitro* was injected into fully grown immature oocytes, which were maintained at the GV-stage or allowed to mature and collected after GVBD or at MII. The length of the poly(A) tail was assessed by RL-PAT using primers that do not amplify cDNA derived from the 3**′**-UTR of endogenous *Slbp* mRNA. (**C**) Line graphs show the length of the cDNA products derived from the injected mRNAs. *wt-Slbp-*3**′**UTR shown in blue, ΔPBE-*Slbp*-3**′**UTR in red. The experiment was performed 2 times.

To understand the basis for the mRNA species-selectivity of CNOT6, we searched for specific sequence elements in the 3ʹ-UTR of these mRNAs. As expected, given that their poly(A) tail lengthens when maturation begins, *Orc6* and *Slbp* contained a putative CPE located near the polyadenylation signal (Fig. 6). U-rich sequences were also present directly upstream or overlapping with the CPEs. Strikingly, both *Orc6* and *Slbp* also contained putative PBE sequences (UGU(A/U)N(AU/UA) lying between the 3′-most CPE and the polyadenylation signal. In contrast, *Actb*, *Ngfr* and *Ccnb1* did not contain PBEs in this position on the 3ʹ-UTR. PBE sequences have previously been associated with deadenylation by the CCR-NOT complex^21,22,47^. To test whether the putative PBEs mediate CNOT6-dependent deadenylation, the 3′-UTR of *Slbp* mRNA was ligated to the firefly luciferase coding sequence (Fig. 7A). Two constructs were generated: one carrying the wild-type sequence of the *Slbp* 3′-UTR (wt-*Slbp*-3′ UTR), and a second one in which the three putative PBEs were deleted (ΔPBE-*Slbp*-3′UTR). cRNAs prepared using these constructs were individually microinjected into immature oocytes, which were then maintained in the immature condition or allowed to mature, after which the length of the poly(A) tail was determined using RL-PAT.

The wt-*Slbp*-3′UTR construct carried a short poly(A) tail in immature oocytes, which lengthened during the early phase of maturation and then shortened at the completion of maturation (Fig. 7B,C). This pattern mirrors the polyadenylation dynamics observed for endogenous *Slbp* and thus demonstrates that this region of the *Slbp* 3′-UTR is sufficient to dictate the polyadenylation pattern of *Slbp* during oocyte maturation. Deletion of the putative PBE sequences had no detectable effect on the length of the poly(A) tail in immature oocytes. In contrast, deletion of these sequences led to an increase in the length of the poly(A) tail in mature oocytes. These results suggest that the putative PBE sequences are required for the CNOT6-dependent deadenylation of mRNAs during late maturation.

### Lengthening the poly(A) tail is not sufficient to increase mRNA translation or inhibit mRNA degradation

**Figure 8:**
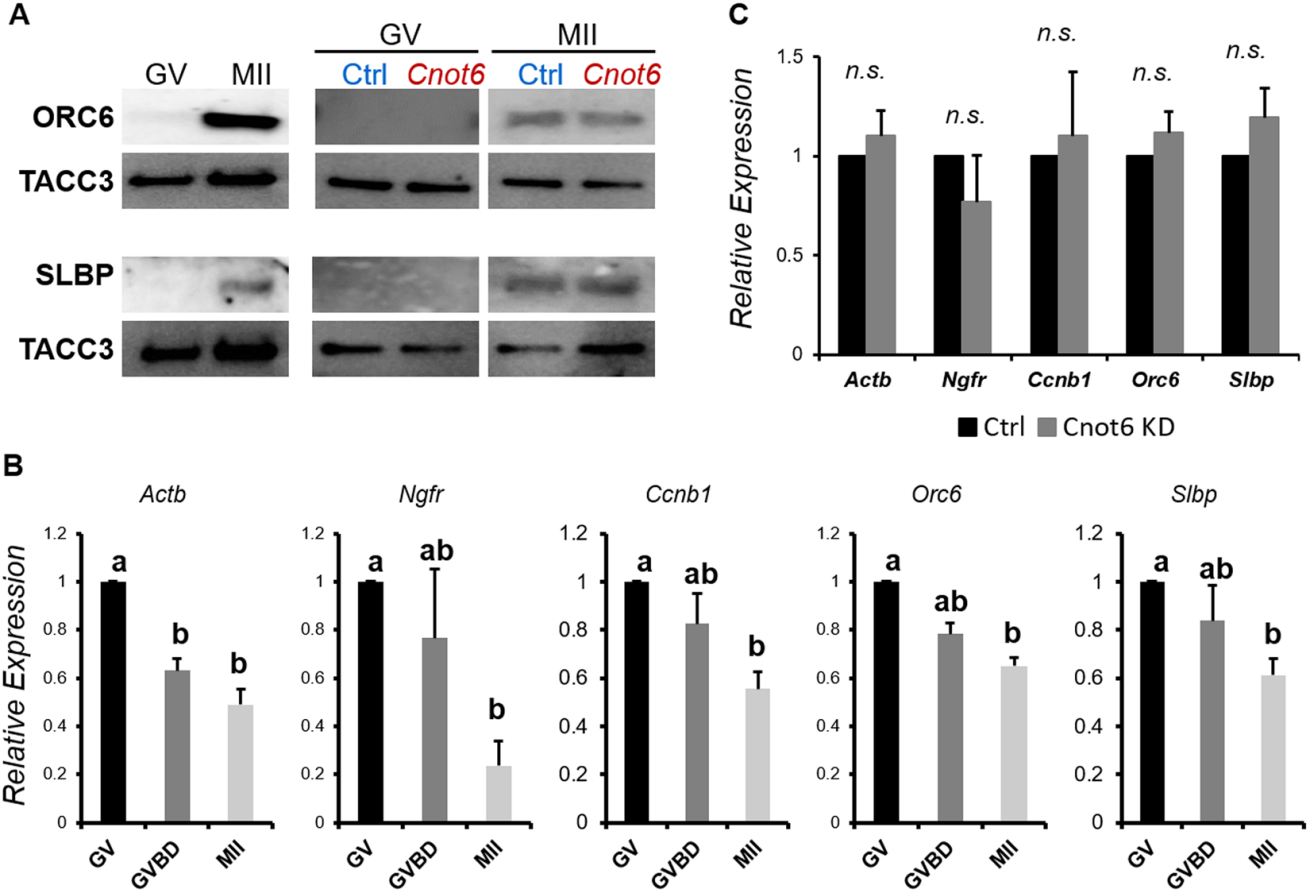
Increasing poly(A) tail-length does not affect mRNA translation or degradation. (**A**) Left: Immunoblot showing relative quantity of ORC6 and SLBP in immature (GV) and mature (MII) oocytes. TACC3 is a loading control. Right: Immature oocytes were injected with control (Ctrl) or *Cnot6* siRNA and either maintained in the immature state (GV) or allowed to mature (MII), then immunoblotted. (**B**) Relative quantity of the indicated mRNAs at different stages of maturation. Data analysed using ANOVA with Tukey HSD; different letters above bars indicate statistically significant difference (*p* < 0.05). (**C**) Relative quantity of the indicated mRNAs in mature oocytes following depletion of CNOT6 as in (**A**). Data analysed using *t*-test.

Because the poly(A) tail has been implicated in the regulation of mRNA translation and degradation, and CNOT6 regulates the poly(A) tail length of *Slbp* and *Orc6* in both immature and mature oocytes, we tested the effect of depleting CNOT6 on the translation and degradation of these mRNAs. Confirming the previous reports^32,33^, we observed that both SLBP and OCT6 increased in abundance during maturation of unmanipulated oocytes (Fig. 8A, left). Depletion of CNOT6 did not, however, increase the amount of either protein in immature or mature oocytes (Fig. 8A, right). Thus, an increase in the length of the poly(A) tail was not sufficient to increase translation of either mRNA at these two stages of oocyte development.

Next, we examined mRNA degradation. We found that the quantities of *Slbp* and *Orc6* declined by about 50% during maturation of unmanipulated oocytes (Fig. 8B). *Actb* and *Ngfr*, whose poly(A) tail shortens during maturation, and *Ccnb1*, whose poly(A) tail lengthens, also declined during this period. These results are consistent with previous reports that oocyte mRNAs begin to be degraded during maturation^3,26,36,48,49^. When we depleted CNOT6, we observed no change in the amount of *Slbp* and *Orc6* in mature oocytes (Fig. 8C). Thus, experimental lengthening of the poly(A) of *Slbp* and *Orc6* did not protect either mRNA from the partial degradation that occurs during maturation.

## Discussion

Previous studies of post-transcriptional regulation of gene expression during oocyte maturation identified two patterns of polyadenylation. Many mRNAs follow what is considered a default pathway – they carry a long poly(A) tail in immature oocytes, which shortens during maturation, when the mRNAs also become translationally suppressed^50^. In contrast, mRNAs that bear one or more CPE in the 3′-UTR within ~200 nt of the polyadenylation signal carry a short poly(A) tail in immature oocytes, which lengthens during maturation when the mRNAs become translationally activated. Here we describe a third pattern of polyadenylation, also recently noted by others^36^, in which mRNAs carry a short poly(A) tail in immature oocytes that becomes transiently lengthened during the early portion of maturation as the mRNAs become translationally activated and then becomes shortened during late maturation.

**Figure 9:**
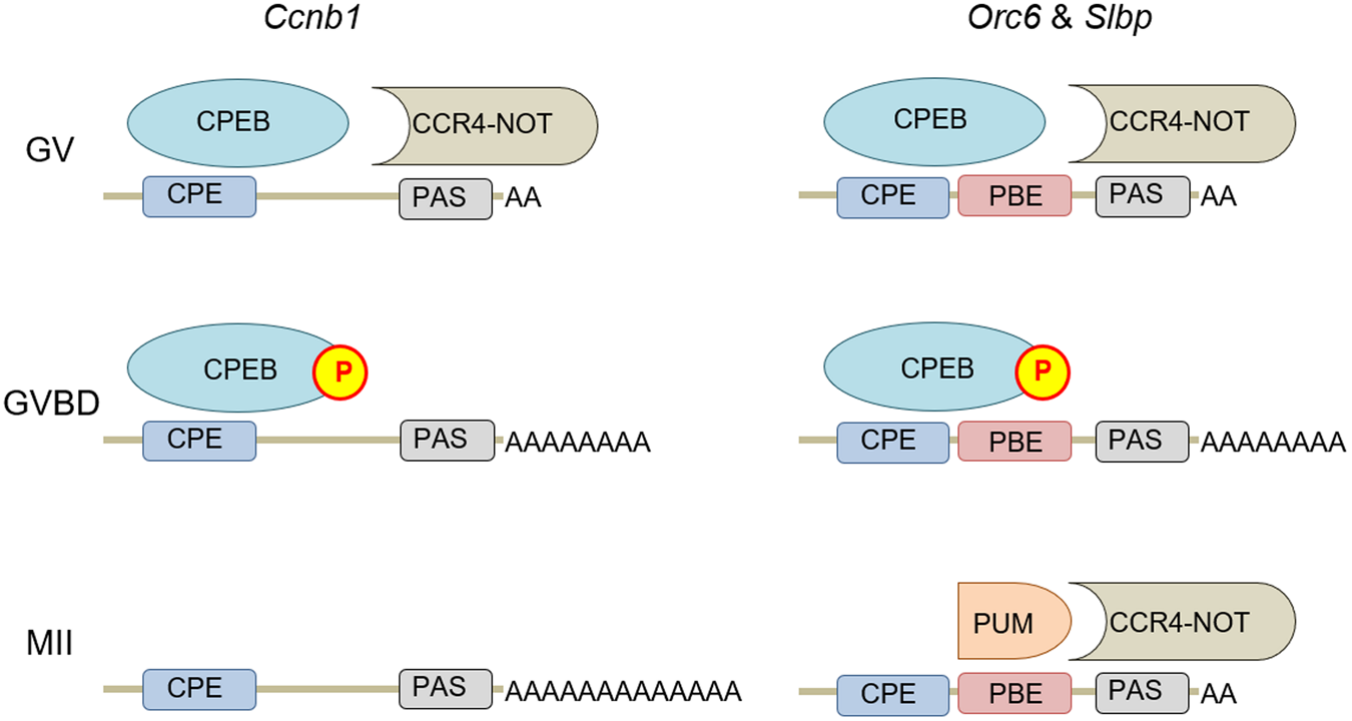
Model for differential poly(A) tail length dynamics during meiotic maturation. In immature (GV) oocytes, the CPE of *Ccnb1*, *Orc6* and *Slbp* provides a binding site for CPEB, which directly or indirectly recruits the CCR4-NOT deadenylase complex. After maturing oocytes undergo GVBD, CPEB becomes phosphorylated. This may inhibit its ability to interact with CCR4-NOT. CPEB nonetheless prevents association of PUM proteins with the nearby PBE sites. In the absence of an associated CCR4-NOT activity, polyadenylation is favoured. Later during maturation, CPEB becomes degraded. This permits PUM proteins to associate with the previously masked PBE sites on *Orc6* and *Slbp*, enabling CCR4-NOT to be recruited to the mRNA. Thus, the mRNA becomes deadenylated. In *Ccnb1*, by contrast, which lacks a PBE in the appropriate location, CCR4-NOT is not recruited and the mRNA continues to be polyadenylated.

Deadenylation of mRNAs can reportedly follow their translation. *Ccnb1* however, is actively translated during maturation, yet does not become deadenylated during late maturation. This suggests that another mechanism is responsible for the late deadenylation, and we provide evidence that putative PBE sequences in the 3ʹ-UTR of *Slbp* and *Orc6* may mediate this process. Figure 9 presents a mechanism by which late deadenylation might occur, based on evidence that both PUF proteins^21,22^ and the CPE-binding protein (CPEB) 1^12,51^ can recruit the CCR4-NOT complex to mRNAs and that CPEB1 becomes phosphorylated during early maturation and is subsequently largely degraded in mature oocytes^25,27,49^. We propose that, in immature oocytes, CPEB recruits CCR4-NOT to the 3′-end of a target mRNA, promoting its deadenylation. During early maturation, CPEB becomes phosphorylated, which impairs its ability to recruit CCR4-NOT. Nonetheless, its sterically hinders binding of PUF proteins to PBE sequences located close to the CPE. In the absence of an associated deadenylase activity, the poly(A) tail of the mRNA lengthens. During late maturation, the degradation of CPEB enables PUF proteins to bind to the PBE present on type III mRNAs and recruit the CCR4-NOT deadenylase complex.

We found that CNOT6 regulated the deadenylation of this subset of mRNAs, but not of other mRNAs, in both immature and mature oocytes. Intriguingly, CNOT7 deadenylase regulates the poly(A) tail-length of other mRNAs in the oocyte^3^. This suggests that different CNOT deadenylases may interact with different mRNAs in the oocyte. In this context, it is worth noting that this type of selectivity has previously been described in somatic cells^11,16,17^. Although the basis for the selectivity remains to be determined, we detected CNOT6 in cortical foci of the oocyte where it frequently co-localizes with 18033 antigens. Previous studies have suggested that cortically associated ribonucleoprotein aggregates in mouse oocytes – particularly those containing 18033 antigens – may be sites of mRNA storage or processing^38,42,52–54^. Although we do not know what proportion of the cellular CNOT6 is present in the foci, it may be speculated that both CNOT6 and its target mRNAs accumulate in cortical foci and that this physical proximity underlies their selective interaction.

Because reducing the quantity of CNOT6 on the oocyte increased the length of the poly(A) tail of *Slbp* and *Orc6*, we were able to study the effect of the longer tail on the activity of these mRNAs. We found that the longer poly(A) did not increase the steady-state level of either encoded protein in immature or mature oocytes. This suggests that an increase in the length of the poly(A) tail, at least of these mRNAs, does not directly drive increased translation. Prior studies, largely utilizing reporter constructs bearing wild-type or mutant CPEs, have nonetheless consistently identified a close link between lengthening of the mRNA poly(A) tail and translational activation^3,36,55–57^. These results may be reconciled by proposing that an increase in poly(A) tail length is a marker of increased translation, but is not an essential part of the mechanism.

We also found that increasing the poly(A) tail length did not prevent the partial degradation of *Slbp* or *Orc6* during maturation. Similarly, increasing the poly(A) tail length of mRNAs regulated by CNOT7 did not prevent their partial degradation during maturation^3^. These results suggest that the mechanism of maternal mRNA degradation during maturation does not depend on deadenylation. The partial degradation of *Ccnb1* during maturation even though it becomes polyadenylated at this time, together with prior studies in *Xenopus*^58^ supports this interpretation. A recent study reported much more extensive mRNA degradation during maturation, and further showed that this degradation was suppressed when BTG1, which promotes deadenylation, was depleted^36^. Although the basis for these differences remains to be elucidated, they may be related to the method used to prepare the cDNA (discussed in^3,26^). In any case, by identifying a distinct pattern of polyadenylation dynamics during maturation and identifying a role for CNOT6 in regulating this process, our results reveal a mechanism by which the activity of specific mRNAs may be selectively regulated in the oocyte.

## Methods

### Mice

All experiments were performed using CD1 mice (Charles River Canada, St.-Constant, QC, Canada) in compliance with the regulations and policies of the Canadian Council on Animal Care and were approved by the Animal Care Committee of the Royal Victoria Hospital (protocol 5991).

### Oocyte collection

Fully grown oocytes containing a visible intact germinal vesicle (GV) and a diameter greater than 70 μm were collected from post-natal day (PD) 19 females at the GV stage. Ovaries were dissected into several fragments and incubated at 37 °C in air in minimal essential medium (MEM, Life Technologies) buffered at pH 7.2 using HEPES (MEM-H), containing sodium pyruvate (0.25 mM, Sigma), penicillin G (63 mg/L, Sigma), streptomycin (50 mg/L, Sigma), and bovine serum albumin (BSA, 1 mg/mL, Sigma). Large follicles were punctured using fine needles. The collection media was supplemented with dibutyryl cyclic AMP (dbcAMP, 0.1 mg/ml, Sigma) to prevent resumption of meiosis. Oocytes were collected using a mouth-controlled micropipette, transferred to fresh medium, and the diameter was measured using an ocular micrometer. To obtain oocytes that had undergone germinal vesicle breakdown (GVBD) and completed maturation to metaphase II, respectively, GV-stage oocytes were transferred to MEM-NAHCO_3_ containing pyruvate, antibiotics and BSA, and incubated at 37 °C in 5% CO_2_ in air for 5 hr or overnight, respectively, to allow meiotic maturation. GVBD oocytes were identified by the absence of the GV and mature oocytes were by the presence of a polar body.

### Reverse transcription-polymerase chain reaction (RT-PCR)

RNA was extracted from oocytes using the ARCTURUS® PicoPure® RNA Isolation Kit (KIT0204; Life Technologies Applied Biosystems) following the manufacturer’s protocol including the optional DNA removal step. RNA samples were then used for reverse transcription and PCR as described^34^. Primers used for PCR are listed in Table 1.

**Table 1:** PCR primer sequences.

Transcript	Sequence
*Cnot6*	F: 5′-GCGACCCGGCAGTTATACGG-3′ R: 5′-AAGTGATGTGGGCAGCCGC-3′
*Cnot6l*	F: 5′-TGGCGCGCTGAATGCAGACTAA-3′ R: 5′-TAGCTGGAAGAGCCGGCCAAGT-3′
*Cnot7*	F: 5′-CCCCGGTGGCTTTCGTCCTC-3′ R: 5′-CACACCGGTCCCTACACGCG-3′
*Cnot8*	F: 5′-ACAGGGGTGGCCCAGAAGCA-3′ R: 5′-TGGTGGCAGGAGGCAGCAGA-3′
*Pan2*	F: 5′-ATGGCCCGTGGAGGGCTCAT-3′ R: 5′-CACAGGTGGTGCTCGCCTGG-3′
*Pan3*	F: 5′-ACTGAAGGAGCAGGCGCTGC-3′ R: 5′-TCCCTCCAGAGTCAGGCCGC-3′
*Parn*	F: 5′-AGGACGCTGAGTCCCGACCC-3′ R: 5′-GCTGCTGCACCGTGAACCCT-3′
*Pnldc*	F: 5′-TTGTCGCGGGTGCGGACTTC-3′ R: 5′-CCAGCGTTGTGGCTGGCTCA-3′
*Cnot1*	F: 5′-TAGGGACTCAGGCTATCGCA-3′ R: 5′-ATGAACTTGGTTCAGCGGAC-3′
*Cnot2*	F: 5′-GGCAACCCAACTCCATTAATAAACC-3′ R: 5′-CCCTGGTAATCCATACCCGC-3′
*Cnot3*	F: 5′-CCCTGAATCCCCTACTCGGT-3′ R: 5′-TTGTCCAGCATTCGCAGGAT-3′
Orc6	F: 5′-TGGGAGATGAAGGGTGTGGA-3′ R: 5′-AATGTCCATGTGAGGTGCCC-3′
*Slbp*	F: 5′-ACCCACCAAAGTCAGACACG-3′ R: 5′-CCAGGCAGAGCCACGTATTT-3′
*Actb*	F: 5′-GGCTGTATTCCCCTCCATCG-3′ R: 5′-CCAGTTGGTAACAATGCCATGT-3′
*Luciferase-Slbp*	F: 5′-CCCAAGCTTGATTTAGGTGAC-3′ R: 5′-(T)_*30*_CAGTTAAAGGGTCTTTA-3′

### RNA ligation-mediated polyadenylation test (RL-PAT)

RL-PAT was performed as previously described^27^. Following RNA extraction, an RNA linker was ligated to the 3′-end of the RNA. Following reverse-transcription, the cDNA was subjected to PCR amplification using nested primers corresponding to the mRNA of interest at the 5′-end and the ligated RNA at the 3′-end. Sequences for the RNA linker and the primers used are listed in Table 2. The products were separated using a 15% agarose gel.

**Table 2:** RNA-Linker and RL-PAT primer sequen**c**es.

Transcript	Sequence	Min length (bp)
RNA Linker	5′-P-CGUAGGCUCAGCUCGGAAUC-ddC-3′	
Linker primer	primer 5′-GGATTCCGAGCTGAGCCTACG	
*Actb*	outer primer: 5′-GTTGGTTGGAGCAAACATCC-3′ inner primer: 5′-GGAAGGTGACAGCATTGCTT-3′	273
*Ngfr*	outer primer: 5′-TCCTTGGCCTGTTCTGTTTT-3′ inner primer: 5′-ACAGACTGACTGCCATCCCT-3′	451
*Ccbn1*	outer primer: 5′-AAAAGAATTTGCCCCCAAGT-3′ inner primer: 5′-CAGCATTCCTTTCAATGCCT-3′	348
*Orc6*	outer primer: 5′-TGCAGTCTGTCTGGGAGATG-3′ inner primer: 5′-TACAGACAGTGAGCGTTCC-3′	153
*Slbp*	outer primer: 5′-GCCTTCAGTTGCCACTTTTC-3′ inner primer: 5′-GCTCTGGACAAAGGATGCTAA-3′	347
*Luciferase-Slbp*	outer primer: 5′-AGATCGTGGATTACGTCGCC-3′ inner primer: 5′-GCTCTGGACAAAGGATGCTAA-3′	347

### Quantitative PCR (qPCR)

Following oocyte collection, 4 pg of rabbit globin mRNA (*Hba1*) (Sigma; R1253) was added to each sample tube prior to RNA extraction as an external control. cDNA was then generated, and qPCR performed using primers listed in Table 3 and EvaGreen-SYBR master mix (Montréal Biotech Inc.; MBI-E500). cDNA amplification was performed using a Rotor-Gene 6000 (Montréal Biotech Inc., Montreal, QC) and analysed using software provided by the manufacturer. Fold-changes in target transcript levels were determined by the 2-∆∆CT method, using globin as the normalizer.

**Table 3:** qPCR primer sequences.

Transcript	Sequence
*Actb*	F: 5′-GGCTGTATTCCCCTCCATCG-3′ R: 5′-CCAGTTGGTAACAATGCCATGT-3′
*Ngfr*	F: 5′-GACTCCTTTACCCACGAGGC-3′ R: 5′-TGGTCAGAAGCAAAGGTCCC-3′
*Ccnb1*	F: 5′-CTGCACTTCCTCCGTAGAGC-3′ R: 5′-TGGTGTCCATTCACCGTTGT-3′
*Orc6*	F: 5′-TTTCCATCTCACTGCAGGCAT-3′ R: 5′-AATGGTCCCAATTTCACCCCA-3′
*Slbp*	F: 5′-ATGGCCTGCAGACCTAGAAG-3′ R: 5′-CTGGCCCAGTCAGAACATCT-3′
*Hba1*	F: 5′-GCAGCCACGGTGGCGAGTAT-3′ R: 5′-GTGGGACAGGAGCTTGAAAT-3′

### Immunoblotting

Oocytes were transferred to the base of a plastic microtube containing 10 μl of 2X Laemmli buffer (Biorad 161-0737) and denatured by heating at 100 °C for 5 min. Samples were then used for immunoblotting as previously described^27^. Antibodies used were against CNOT6 (Sigma SAB2100457, 10 μg/mL), CNOT7 (Sigma WH0029883M1, 5 μg/mL), ORC6 (Cell Signalling 4737 S, 1:1000), SLBP (Abnova H00007884-M01, 200 ng/mL), tubulin (Sigma T8203, 10 μg/mL) and TACC3 (Upstate Biotechnology Cat#07-233, 1 ng/µL). Signals were generated using enhanced chemiluminescence (Thermo Scientific 80196) and detected using film and a phosphorimager (Amersham Storm). Intensities were quantified using ImageJ (National Institutes of Health).

### Whole-cell immunofluorescence

Oocytes were fixed for 20 min in a solution of 2% para-formaldehyde (Fisher Scientific 04042) in phosphate buffered saline (PBS) containing 0.1% Triton X-100 (ACROS 9002-93-1) and stored in a blocking solution (3% BSA + 0.1% Triton X-100 in PBS). Fixed oocytes were incubated overnight at 4 °C on a shaker in primary antibody diluted appropriately in the blocking solution. Following two 5-min washes in blocking solution on a shaker at room temperature, oocytes were then incubated in secondary antibody diluted in the blocking solution for 1 hr at room temperature. DRAQ5 (5 μM, New England Biolabs 4084S) and Phalloidin-TRITC (5 μg/mL, Sigma P1951) were added to the secondary antibody solution to label the nucleus and the actin-cytoskeleton, respectively. Oocytes were then washed twice with blocking solution, and mounted onto slides, in a PBS drop covered with mineral oil using spacers (GBL654008, Sigma) to avoid crushing the samples. Primary antibodies were: CNOT6 (1 μg/mL, Sigma SAB2100457), 18033 (1:10000, a kind gift of Dr. M. Fritzler), and LSM14A (1:500, a kind gift of Dr. J. Lykke-Anderson). Secondary antibodies were Alexa 488-conjugated goat anti-rabbit antibody (10 μg/mL, Life Technologies A11008), used to detect CNOT6 and LSM14A, and Dylight 549-conjugated goat anti-human antibody, used to detect GW182. Preparations were mounted on glass slides and imaged using a Zeiss CLSM 510 confocal scanning laser microscope.

Cortical signal intensity of CNOT6 following siRNA injection was quantified using the CLSM 510 software. Two concentric circles were drawn outside and inside the oocyte cortical region, respectively. The areas outside the outer circle and inside the inner circle were masked and the signal within the remaining area (encompassed by the outer but not inner circle) was measured. The mean signal intensity was then calculated for each experimental group and compared using the t-test.

Co-localization of CNOT6 and GW182 was analyzed using ImageJ. The Pearson correlation coefficient (Rr) and Mander’s overlap coefficient (R) were calculated to determine the correlation of incidence and percentage overlap, respectively, of the two signals.

### Immunohistofluorescence

Ovaries were collected from 6-week old mice and fixed overnight in 4% para-formaldehyde at 4 °C. They were then washed three times for 15 min each in PBS and then stored at 4 °C. The specimens were then embedded in paraffin, sectioned, and mounted on glass slides. Antigen retrieval and antibody staining were performed as described^59^.

### siRNA microinjection

Fully grown oocytes were transferred in groups of 15 to a pre-warmed 25-µl drop of MEM-HEPES supplemented with dbcAMP in a 35-mm plastic culture dish and covered with mineral oil. The dish was then moved to an inverted microscope (Zeiss; Observer.Z1 AX70) equipped with micromanipulators (Ependorff, Transferman 4r). Using a micro-injector (Medical Systems Corp; PL-100) as previously described^27^, 5 µl of an siRNA solution was injected into the cytoplasm of each oocyte. The following siRNAs were used: *Cnot6* (20 μM, Thermo Scientific 1299001), *Rspo1* (20 μM, Life Technologies MSS247584). *Rspo1* is not detectable in oocytes (S. El-Hayek, unpublished). Following injection, oocytes were incubated overnight in MEM supplemented with dbcAMP as described^60^. Oocytes to be analyzed at the GV stage were then incubated an additional day in the presence of dbcAMP. Oocytes to be analyzed at metaphase II were transferred to dbcAMP-free MEM and incubated an additional day. The oocytes were then used either for whole-cell immunofluorescence or for RNA analysis by quantitative PCR or RL-PAT.

### cRNA injections

Luciferase-Slbp 3′-UTR constructs were generated using the previously describe methods^27^. The entire *Slbp* 3′-UTR (starting from the stop codon to the end of the polyadenylation signal) was amplified using primers incorporating a *XhoI* site. The amplified product was then inserted into a pCS2+ plasmid (gift from Dr. M. Featherstone), downstream of a Luciferase ORF at the XhoI site. Alternatively, the plasmid was also PCR-amplified using primers that excluded the three putative PUF- binding elements (PBEs) located 92 nt–104 nt upstream of the polyadenylation signal, modelled after previously described methods. This generated the ΔPBE-*Slbp*-3′UTR construct that lacked the three putative PBEs in the *Slbp* 3′-UTR. The constructs were then amplified within a product that also included the SP6 promoter from the plasmids, using primers that also incorporated a 30-nt long poly(A) tail at their 3′-end (Table 1). Both constructs were transcribed *in-vitro* to yield cRNAs with a 30-nt long poly(A) tail using the mMessage mMachine SP6 Transcription kit (Invitrogen; AM1340). cRNAs (100 nM) were then individually injected in GV oocytes.

### Data availability

The datasets generated during and/or analysed during the current study are available from the corresponding author on reasonable request.

## Electronic supplementary material

Supplementary information
