# Supplementary material for: CNOT6 regulates a novel pattern of mRNA deadenylation during oocyte meiotic maturation
Source: Sci Rep. 2018 May 1;8:6812. doi: 10.1038/s41598-018-25187-0 (PMC5931610; doi:10.1038/s41598-018-25187-0)

CNOT6 regulates a novel pattern of mRNA deadenylation during oocyte meiotic maturation

K.-F. Vieux and H. J. Clarke

SUPPLEMENTARY INFORMATION

A. Full-length gels corresponding to Figure 1.

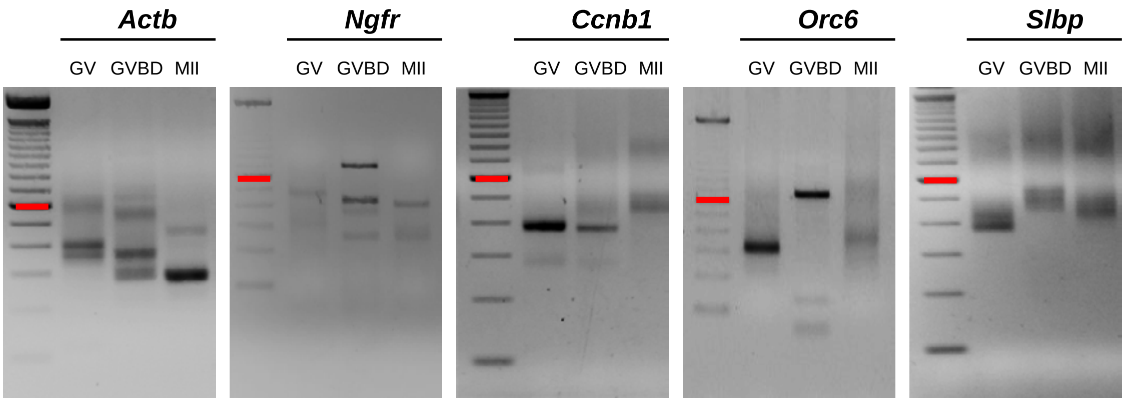

B. Full-length gels corresponding to Figure 2A. Red line in lane L is 600 nt marker. Lanes correspond to oocytes at early, mid- and fully grown stages, and mature eggs. Lanes in red are shown in the manuscript.

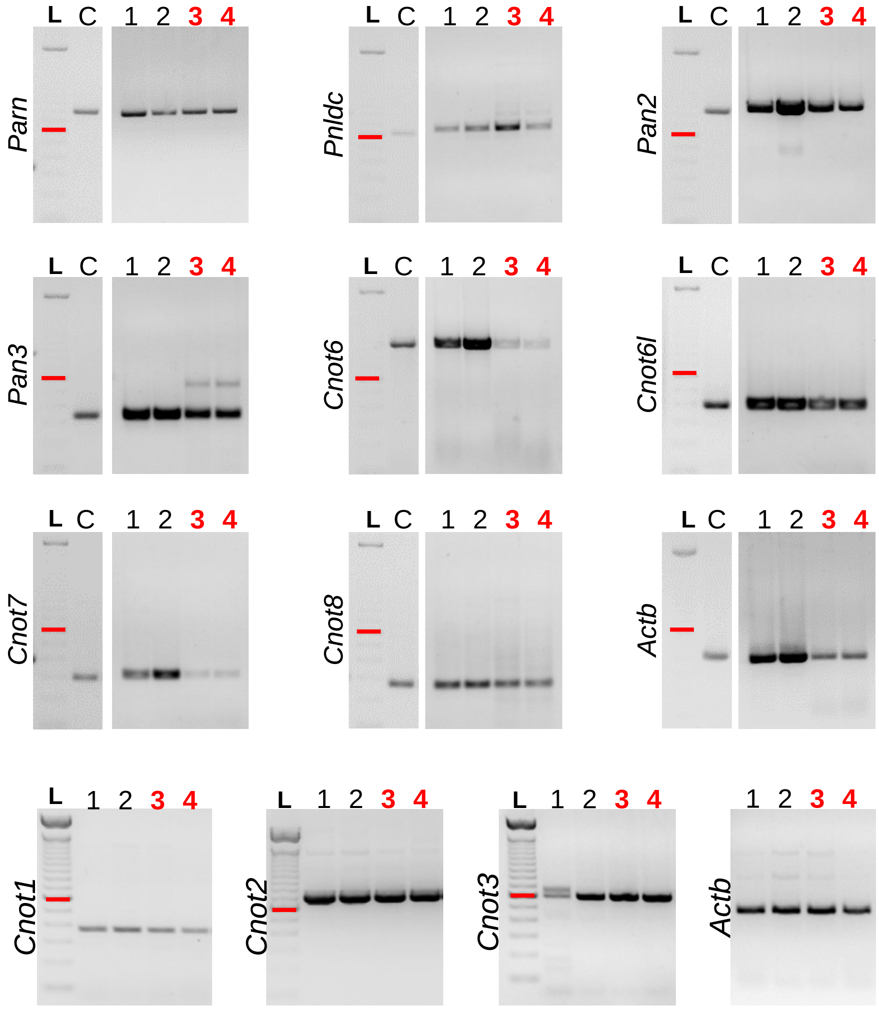

C. Full-length gels corresponding to Figure 2B. Left: Blot probed for CNOT6 and then for tubulin. The same blot was cut as shown to blot for CNOT7. Right: Sample was divided into two aliquots that were run on separate gels. One was blotted for CNOT6, the other for tubulin. This blot is shown in the manuscript.

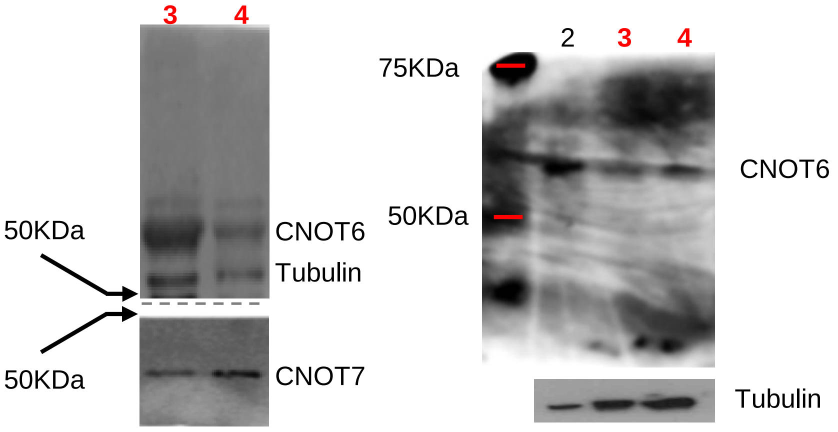

D. Full-length gels corresponding to Figure 5C. Note the images are displayed in reverse black-white in the manuscript. Note lanes are switched in the *Orc6*-GV panel

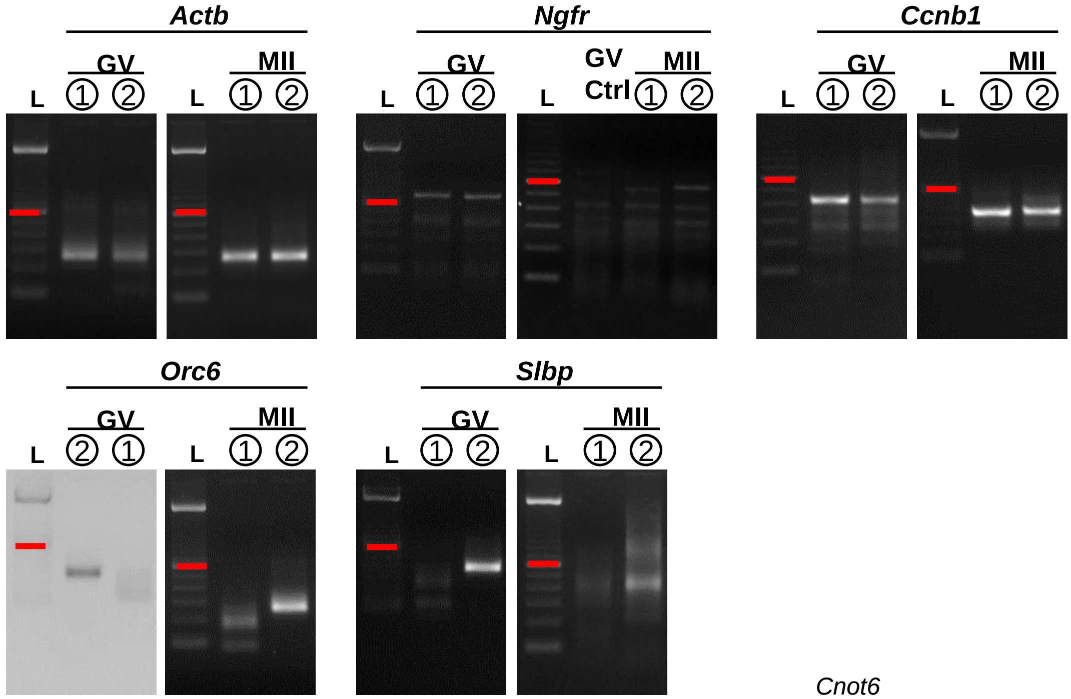

E. Full-length gel corresponding to Figure 5A.

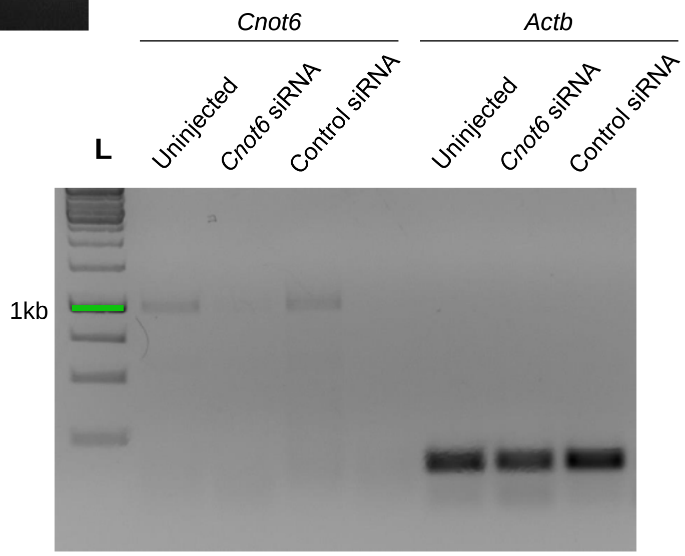

F. Full-length gels corresponding to Figure 7B.

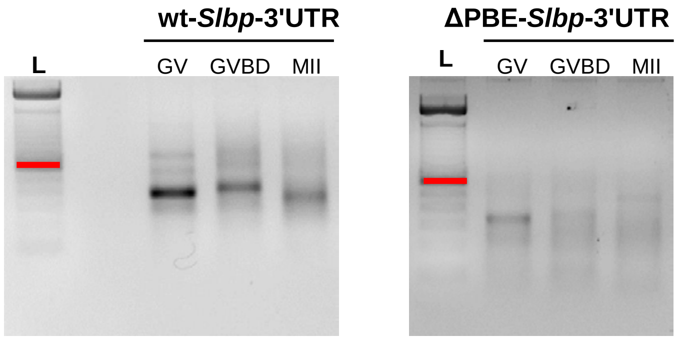

G. Full-length gels corresponding to Figure 8A (ORC6). Dotted line shows where gels were cut before immunoblotting with indicated antibody.

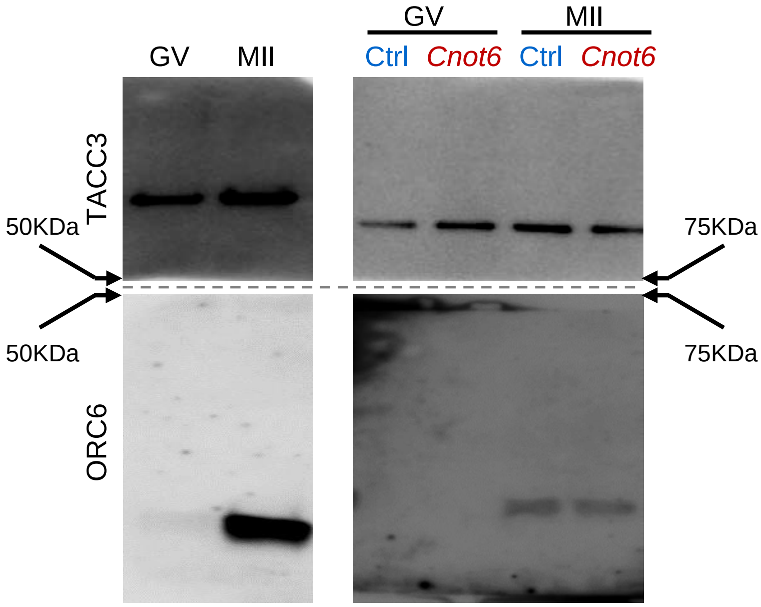

H. Full-length gels corresponding to Figure 8A (SLBP). Dotted line shows where gels were cut before immunoblotting with indicated antibody.

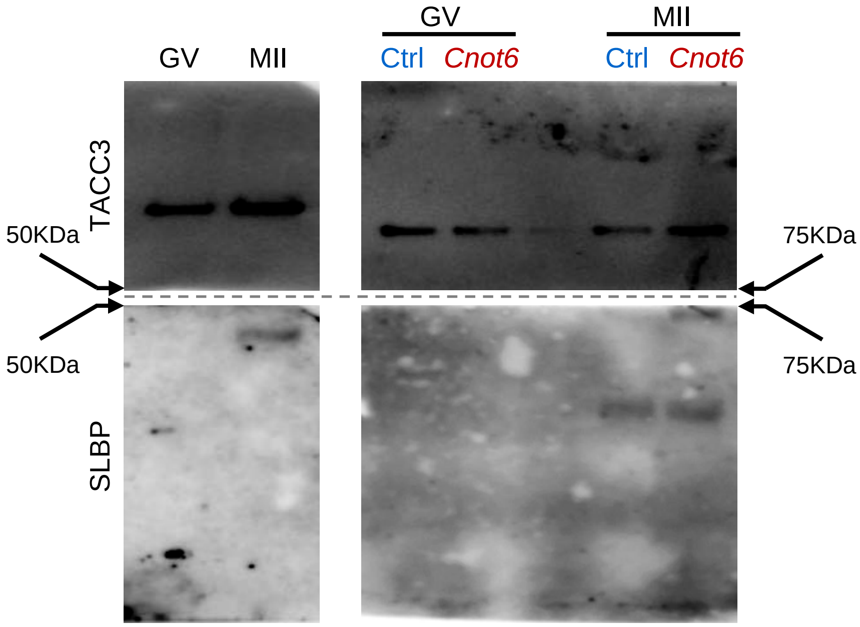

Supplement: Supplementary file 1 — Supplementary information [file 41598_2018_25187_MOESM1_ESM.pdf]
